# Supplementary material for: Prognostic impact of misdiagnosis of cardiac channelopathies as epilepsy
Source: PLoS One. 2020 Apr 16;15(4):e0231442. doi: 10.1371/journal.pone.0231442 (PMC7161979; doi:10.1371/journal.pone.0231442)
Supplement: S1 Methods — (DOCX) [file pone.0231442.s001.docx]

SUPPLEMENTAL METHODS

Genetic Evaluation

Blood samples from probands were subjected to automated genomic DNA purification (QIAsymphony SP®, Qiagen). Library preparation was carried out using the SureSelectXT Reagent library preparation kit (Agilent) for Illumina paired-end multiplexed sequencing method. Enrichment was performed using a custom Clinical Research Exome (Agilent) which selectively captures coding regions and adjacent intronic areas for the selected genes. After cluster generation on a cBot (Illumina), captured DNA was sequenced on the Illumina HiSeq 1500 platform. Clinically relevant variants and low-coverage regions were tested in parallel by standard Sanger sequencing. Bioinformatics analysis was performed by means of a custom pipeline that included software such as NovoAlign, GATK, SAMtools and Bcftools for variant calling and genotyping. First, the NGS reads were subjected to quality control checks to remove any low quality reads. Then the reads were mapped (aligned) to hg19. ANNOVAR (http://annovar.openbioinformatics.org) software was used to functionally annotate detected variants.

The list of validated variants was further trimmed by omitting those reported in dbSNP, or present in international databases (1000 Genomes project, ExAC, gnomAD) with a MAF ≥ 1%. Variant deleteriousness was predicted using different web-based tools: Polyphen-2, SIFT, MutationTaster and DANN. To predict splice-site variant effect, three different software tools were used: NNSplice, MaxEntScan, and HSFHuman Splicing Finder.
