# Supplementary material for: Prognostic impact of misdiagnosis of cardiac channelopathies as epilepsy
Source: PLoS One. 2020 Apr 16;15(4):e0231442. doi: 10.1371/journal.pone.0231442 (PMC7161979; doi:10.1371/journal.pone.0231442)
Supplement: S1 Table — (DOCX) [file pone.0231442.s002.docx]

| *CACNA1C* | *CALM1* | *CALM2* | *CALM3* | *CASQ2* | *DES* | *DSC2* | *DSG2* |
| --- | --- | --- | --- | --- | --- | --- | --- |
| *FLNC* | *JUP* | *KCNE1* | *KCNE2* | *KCNH2* | *KCNJ2* | *KCNQ1* | *LMNA* |
| *PKP2* | *PLN* | *PRKAG2* | *RYR2* | *SCN5A* | *TNNC1* | *TNNI3* | *TNNT2* |
| *MYH7* | *NKX2-5* | *CACNA2D1* | *CACNB2* | *CAV3* | *FHL2* | *GAA* | *GJA5* |
| *GPD1L* | *HCN4* | *IRX3* | *KCNA5* | *KCND3* | *KCNE3* | *KCNE5* | *KCNJ5* |
| *MYH6* | *PITX2* | *SCN1B* | *SCN2B* | *SCN4B* | *SLC22A5* | *SNTA1* | *TBX5* |
| *ANK2* | *CACNA1D* | *ACTC1* | *GLA* | *AKAP9* | *GNB2* | *KCNJ8* | *TECRL* |
| *LAMP2* | *TMEM43* | *TNNI3K* | *TRDN* | *TRPM4* | *TTR* | *ABCC9* | *ANK3* |
| *CAVIN1* | *CAVIN4* | *CDH2* | *FGF12* | *GATA5* | *GJA1* | *GREM2* | *KCND2* |
| *KCNK17* | *KCNK3* | *LDB3* | *MYBPHL* | *NKX2-6* | *NOS1AP* | *NPPA* | *PPA2* |
| *RANGRF* | *SCN10A* | *SCN3B* | *SLMAP* | *SYNE2* | *TMEM175* | *TPM1* | *ZFHX3* |

***Table S1. Genes included in the NGS panel***
