# Supplementary material for: Prognostic impact of misdiagnosis of cardiac channelopathies as epilepsy
Source: PLoS One. 2020 Apr 16;15(4):e0231442. doi: 10.1371/journal.pone.0231442 (PMC7161979; doi:10.1371/journal.pone.0231442)
Supplement: S2 Table — (DOCX) [file pone.0231442.s003.docx]

**Table S2. Mutations identified in patients diagnosed with a cardiac channelopathy.**

| **PATIENTS WITH EPILEPSY DIAGNOSIS** | | | | |
| --- | --- | --- | --- | --- |
| **Subject** | **Diagnosis** | **Gene** | **Protein** | **Variant information** |
| Patient 1 | LQTS | *KCNH2* | Potassium voltage-gated channel subfamily H member 2 | NM_000229.1:p.Glu637Gly  NM_000238.3:c.1790A>R  NC_00007.13g.150648571T>C |
| Patient 2 | LQTS | *KCNH2* | Potassium voltage-gated channel subfamily H member 2 | NP_000229.1:p.Gly572Asp  NM_000238.3:c.1715G>A  NC_000007.13:g.150648776C>T |
| Patient 3 | BrS | *SCN5A* | Sodium channel protein type 5 subunit alpha | NP_932173.1:p.Arg893His  NM_198056.2:c.2678G>A  NC_000003.11:g.38627291C>T |
| Patient 4 | LQTS | *KCNH2* | Potassium voltage-gated channel subfamily H member 2 | NP_000229.1:p.Ile30Phe  NM_000238.3:c.88A>T  NC_000007.13:g.150672018T>A |
| Patient 5 | CPVT | *RYR2* | Ryanodine receptor 2 | NP_001026.2:p.Ala2387Thr  NM_001035.3:c.7159G>A  NC_000001.10:g.237804240G>A |
| Patient 6 | LQTS | *KCNQ1* | Potassium voltage-gated channel subfamily KQT member 1 | NP_000209.2:p. p.Arg366Gln  NM_000218.2:c.1097G>A  NC_00011.10:g.2585276G>A |
| **PATIENTS WITHOUT EPILEPSY DIAGNOSIS** | | | | |
| **Subject** | **Diagnosis** | **Gene** | **Protein** | **Variant information** |
| Patient 7 | LQTS | *KCNQ1* | Potassium voltage-gated channel subfamily KQT member 1 | Unavailable |
| Patient 8 | LQTS | *KCNH2* | Potassium voltage-gated channel subfamily H member 2 | NP_000229.1:p.Gly628Ser  NM_000238.3c.1882 G>A  NC_000007.13:g-150648599C>T |
| Patient 9 | LQTS | *KCNQ1* | Potassium voltage-gated channel subfamily KQT member 1 | NP_000209.2:p.Ser644Metfs*22  NM_000218.2:c.1931delG  NC_000011.9:g.2869133delG |
|  | LQTS | *KCNH2* | Potassium voltage-gated channel subfamily H member 2 | NP_000229.1:p.Val115Met  NM_000238.3:c.343G>A  NC_000007.13:g.150656789C>T |
| Patient 10 | LQTS | *KCNH2* | Potassium voltage-gated channel subfamily H member 2 | NP_000229.1:p.Pro1034fs  NM_00238.3:c.3100_3109del  NC_000007.13:g.150644463_150644473del |
| Patient 11 | LQTS | *KCNQ1* | Potassium voltage-gated channel subfamily KQT member 1 | NP_000209.2:p.Ala341Val  NM_000218.2:c.1022C>T  NC_000011.9:g.2604765C>T |
| Patient 12 | CPVT | *RYR2* | Ryanodine receptor 2 | NP_001026.2:p.Ser4006Thr  NM_001035.2:c.12016T>A  NC_000001.10:g.237947028T>A |
| Patient 13 | CPVT | *RYR2* | Ryanodine receptor 2 | NP_001026.2:p.Leu488Ile  NM_001035.2:c.1462C>A  NC_000001.10:g.237617860C>A |
| Patient 14 | LQTS | *KCNQ1* | Potassium voltage-gated channel subfamily KQT member 1 | NP_000209.2:p.Leu191Gln  NM_000218.2:c.572T>A  NC_000011.9:g.2591952T>A |
| Patient 15 | LQTS | *SCN5A* | Sodium channel protein type 5 subunit alpha | NP_932173.1:p.Arg1644His  NM_198056.2:c.4931G>A  NC_000003.11g.38592932C>T |
| Patient 16 | BrS | *SCN5A* | Sodium channel protein type 5 subunit alpha | NP_001092874.1:p.Tyr1449Cys  NM_0010994.1:c.4346A>G  NC_000003.11:g.38598023T>C |
| Patient 17 | LQTS | *KCNQ1* | Potassium voltage-gated channel subfamily KQT member 1 | NP_000209.2:p.Ala344Val  NM_000218.2:c.1031C>T  NC_000011.9:g.2604774C>T |
| Patient 18 | CPVT | *CASQ2* | Calsequestrin-2 | NP_001223.2:p.Arg33*  NM_001232.3:c.97C>T  NC_000001.11:g.225768445G>A |
| Patient 19 | CPVT | *CASQ2* | Calsequestrin-2 | NP_001223.2:p.(Gly127=)  NM_001232.3:c.381C>T  NC_000001.10:g.116283388G>A |
| Patient 20 | CPVT | *CALM2* | Calmodulina | NP_001734.1:p.Asn98Ser  NM_001743.4:c.293A>G  NC_000002.11:g.47388990T>C |
| Patient 21 | CPVT | *CALM2* | Calmodulina | NP_001734.1:p.Asn98Ser  NM_001743.4:c.293A>G  NC_000002.11:g.47388990T>C |
| Patient 22 | CPVT | *CASQ2* | Calsequestrin-2 | NP_001223.2:p.(Gly127=)  NM_001232.3:c.381C>T  NC_000001.10:g.116283388G>A |
| Patient 23 | CPVT | *RYR2* | Ryanodine receptor 2 | NP_001026.2:p.Asp4195Asn  NM_001035.2:c.12583G>A  NC_000001.10:g.237947595G>A |
| Patient 24 | CPVT | *RYR2* | Ryanodine receptor 2 | NP_001026.2:p.Lys337Asn  NM_001035.2:c.1011A>C  NC_000001.10:g398923A>C |
| Patient 25 | LQTS | *SCN5A* | Sodium channel protein type 5 subunit alpha | NP_001092874.1:p.Gly1329Ser  NM_001099404.1:c.3985 G>A  NC_000003.12:g.38560407C>T |
| Patient 26 | CPVT | *RYR2* | Ryanodine receptor 2 | NP_001026.2:p.Leu4915Trp  NM_001025.2:c.14744T>G  NC_000001.10:g.237993918T>G |
| Patient 27 | CPVT | *RYR2* | Ryanodine receptor 2 | NP_001026.2:p.His4742Gln  NM_001025.2:c.14226C>G  NC_000001.10:g237969511C>G |
| Patient 28 | LQTS | *KCNJ2* | Inward rectifier potassium channel 2 | NP_000882.1:p.Arg82Trp  NM_000891.2:c.244C>T  NC_000017.10:g68171424C>T |
| Patient 29 | CPVT | *RYR2* | Ryanodine receptor 2 | NP_ 001026.2:p.Arg176Gln  NM_001035.2:c.527G>A  NC_000001.10:g.237540686G>A |
| Patient 30 | LQTS | *KCNH2* | Potassium voltage-gated channel subfamily H member 2 | NP_000229.1:p.Ala614Val  NM_000238.3:c.1841C>T  NC_000007.13:g.150648640G>A |
| Patient 31 | LQTS | *KCNJ2* | Inward rectifier potassium channel 2 | NP_000882.1:p.Cys122Tyr  NM_00891.2:c.365G>A  NC_000017.10:g.68171545G>A |
